# How the acceleration phase influences energy flow and the resulting joint moments of the throwing shoulder in the deceleration phase of the javelin throw

**DOI:** 10.3389/fspor.2024.1445455

**Published:** 2024-09-27

**Authors:** Hans-Peter Köhler, Maximilian Schödlbauer, Maren Witt

**Affiliations:** Department of Biomechanics in Sports, Leipzig University, Leipzig, Germany

**Keywords:** optimization, injury prevention, athletics, modelling, inverse dynamics

## Abstract

**Introduction:**

The throwing motion in the javelin throw applies high loads to the musculoskeletal system of the shoulder, both in the acceleration and deceleration phases. While the loads occurring during the acceleration phase and their relationship to kinematics and energy flow have been relatively well investigated, there is a lack of studies focusing the deceleration phase. Therefore, the aim of this study is to investigate how the throwing arm is brought to rest, which resultant joint torques are placed on the shoulder and how they are influenced by the kinematics of the acceleration phase.

**Methods:**

The throwing movement of 10 javelin throwers were recorded using a 12-infrared camera system recording at 300 Hz and 16 markers placed on the body. Joint kinematics, kinetics and energy flow were calculated between the touchdown of the rear leg and the timepoint of maximum internal rotation after release +0.1 s. Elastic net regularization regression was used to predict the joint loads in the deceleration phase using the kinematics and energy flow of the acceleration phase.

**Results:**

The results show that a significant amount of energy is transferred back to the proximal segments, while a smaller amount of energy is absorbed. Furthermore, relationships between the kinematics and the energy flow in the acceleration phase and the loads placed on the shoulder joint in the deceleration phase, based on the elastic net regularized regression, could be established.

**Discussion:**

The results indicate that the loads of the deceleration phase placed on the shoulder can be influenced by the kinematics of the acceleration phase. For example, an additional upper body forward tilt can help to increase the braking distance of the arm and thus contribute to a reduced joint load. Furthermore, the energy flow of the acceleration phase can be linked to joint stress. However, as previously demonstrated the generation of mechanical energy at the shoulder seems to have a negative effect on shoulder loading while the transfer can help optimize the stress. The results therefore show initial potential for optimizing movement, to reduce strain and improve injury prevention in the deceleration phase.

## 1 Introduction

Javelin throwing is a demanding activity for the throwing arm and shoulder. In Javelin throwing release velocities over 30 ms^−1^ are achieved in competition (1). To accelerate the implement, mechanical energy must be transferred through the shoulder and throwing arm, while the energy is generated by the larger proximal segments by the acceleration of the thrower and the implement in the run-up phase. The resulting loads placed on the joints of the throwing arm, which are necessary to transfer the energy through the kinetic chain exceed the requirements in baseball due to the much heavier implement, although significantly higher throwing speeds are achieved when pitching (2). After the release, the body of the athlete must be brought to rest to avoid crossing the foul line. Therefore, also the throwing arm, which is accelerated to high velocities in the acceleration phase, must be decelerated. The remaining kinetic energy must be dissipated, for which different options are possible. In general, energy can be produced or absorbed by the muscles, energy can be stored and recoiled by elastic elements of the muscle-tendon unit and the ligaments, and energy can be transferred from proximal to distal joints and vice versa via the biarticular muscles and/or gravitational and inertial forces (3). However, the deceleration motions put the muscles of the rotator-cuff, its tendons, and the capsule of the humerus of the throwing shoulder under high stress, as not only must the motion be stopped, but the humeral head must also be prevented from distraction. This phase was attributed to tensile failure and resulting rotator cuff tears due to the high loads necessary to decelerate the arm (4–6).

While the resultant joint torque placed on the shoulder have been investigated frequently during the acceleration phase, especially in baseball throwing, only little is known about the demands of the deceleration/follow-through phase. The kinematic variables that the athlete must deal with have been particularly well researched. For instance, shoulder internal rotation velocities up to 8,000°/s, reached shortly after release and linear velocities of the hand near the release speed of the implement must be decelerated in baseball pitching (7, 8). In javelin throwing, the (angular) velocities that have to be dealt with are lower due to the higher mass of the implement and the associated lower release speeds (9). Therefore, the remaining energy of the segments, which must be dissipated, should also be lower. In baseball, Fleisig et al. (10) summarized the resultant joint torques placed on the shoulder in the deceleration phase, which the muscles of a joint have to balance and are a necessary basis for energy transfer and absorption, with up to 83 ± 26 Nm for shoulder adduction, 97 ± 25 Nm for shoulder horizontal abduction (extension) and 7 ± 5 Nm for external shoulder rotation. To the best of our knowledge, this overview is complete as the focus in recent years has been largely on the requirements of the acceleration phase. Which factors influences the resultant joint torques at the shoulder in decelerating motions is unknown. Only Solomito et al. (11) have calculated the influence of kinematics, i.e., the elbow angle on the resultant joint torques of the elbow valgus torque in the deceleration phase. They concluded that greater flexion angles of the elbow during pitching raises the resultant joint torques placed on the elbow in the deceleration phase. It must therefore be assumed for javelin throwing that the kinematics of the acceleration phase influence the resultant joint torques placed on the shoulder in the deceleration phase, as different joint angles lead to different lever arms and moments of inertia.

However, not only the kinematics and kinetics are of interest, so is also the energy flow (EF) between the segments of the throwing arm caused by gravitational and inertial forces. Due to the requirement of high rates of energy transfer and large amount of mechanical energy transferred to the distal segment in the acceleration phase, higher release speeds also mean that segments must be accelerated to higher velocities (2, 9). Therefore, higher speeds must also be decelerated, and the remaining energy dissipated. But how this is done has not yet been thoroughly examined. Only Wasserberger et al. (12) investigated the EF of the deceleration phase in baseball pitching by calculating the EF due to resultant moments and torques or in other words due to gravitational and inertial forces. They showed, that large amounts of energy are transferred proximally through the elbow (168 ± 72 J) and shoulder (129 ± 61 J) joint, when decelerating the throwing arm. They were also able to show that the shoulder absorbs a significant amount of mechanical energy (79 ± 36 J). The technique of analyzing the EF due to gravitational and inertial forces and torques used by Wasserberger et al. (12) has recently become very popular. This technique can be used to study the EF between adjacent segments and enables the calculation of the transfer, generation, and absorption of mechanical energy at the connecting joint. Furthermore, it is also possible to determine the contributions of rotational and linear kinetics which arise from resultant joint torques and forces, respectively (13). EF analysis has been used in different sports like tennis (14), baseball (15, 16), table tennis (17) and javelin throwing (2) for different regions of the body. The various sports have shown that EF analysis enables the investigation of mechanical patterns and thus expands the understanding of the movements in the kinematic chain.

While the focus of studies has mostly been on the acceleration phase, the deceleration phase has been much less frequently studied. Furthermore, the influence of kinematics and energy flow in the acceleration phase on the resultant joint torque and energy flow in the deceleration phase has not been investigated to date in javelin throwing. Therefore, the aim of the study was to investigate (i) how the remaining mechanical energy of the throwing arm is dissipated through gravitational and inertial forces via the shoulder, (ii) which resultant joint torques are placed on the shoulder and (iii) how the resultant joint loads at the shoulder are influenced by the kinematics and energy flow of the acceleration phase.

## 2 Methods

### 2.1 Participants

Ten right-handed javelin throwers (body height: 189.2 ± 7.2 cm; body mass: 92.4 ± 9.3 kg; age: 21.8 ± 3.6 years; personal best: 78.23 ± 11.38 m) participated in the study. At the timepoint of the investigation, all athletes were free from injury. Leipzig University Ethics Committee approved the investigation (ethical approval nr: 462/18-EK). Prior to the investigation, all participants gave written informed consent to participate in the study. The study was conducted according to the declaration of Helsinki.

### 2.2 Material and experimental protocol

**Figure 1:**
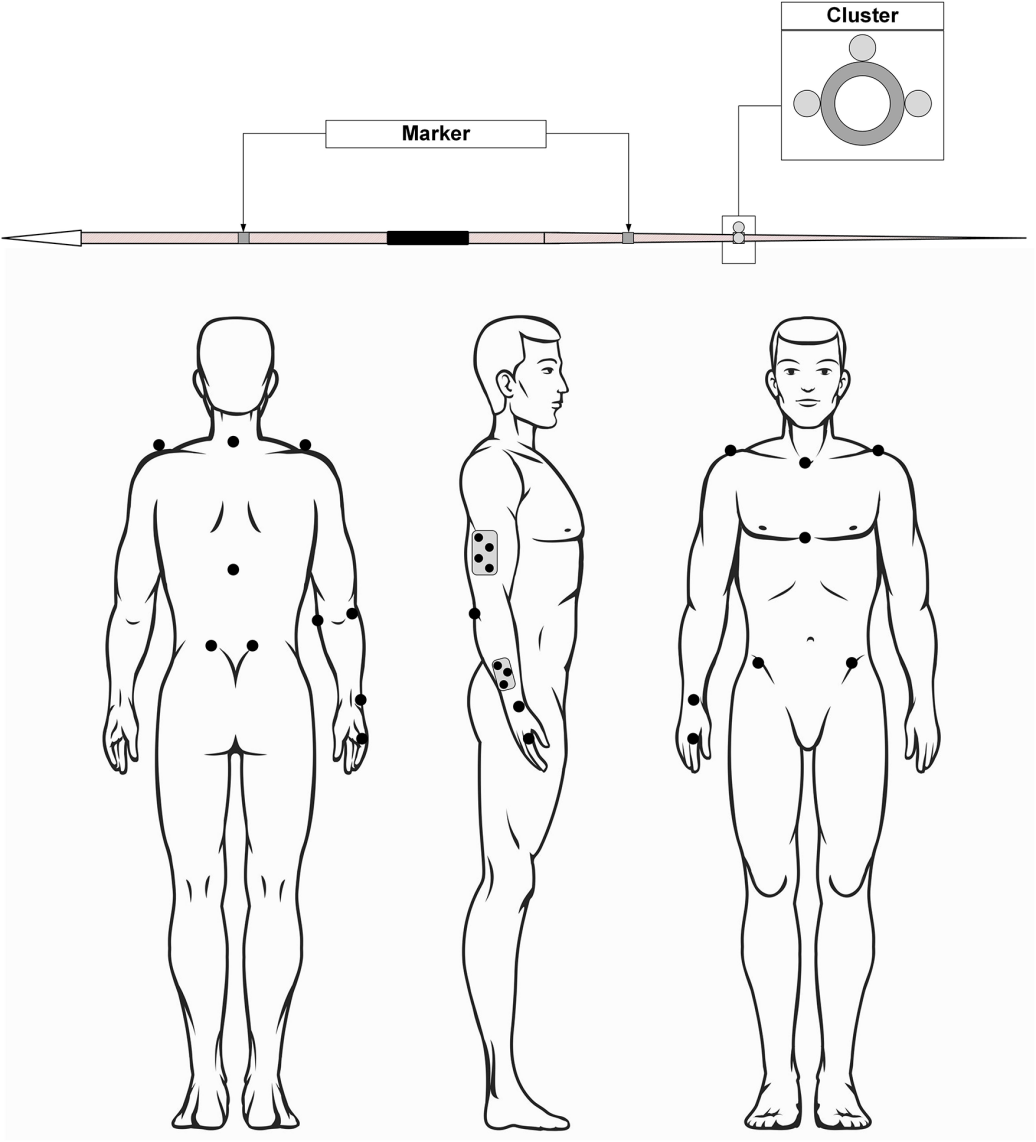
Marker placement.

Sixteen markers (metacarpophalangeal joint of the 2nd and 5th finger; ulnar and radial styloid; lateral and medial epicondyle of the humerus; left and right acromion; 7th cervical vertebrae and 12th thoracic vertebrae; processus xiphoideus; incisura jugularis; left and right spina iliaca posterior superior; left and right spina iliaca anterior superior) and two clusters (upper arm, forearm) were placed on anatomical landmarks off each subject in order to record the movements of the thrower's torso and upper extremities (Figure 1). The javelin (GETRA Kinetic, 800 g, 70 m) had five markers attached to it. It was modified for indoor use by replacing the sharp metal tip with a dull carbon one. As part of the indoor investigation, the athletes threw the javelin into a safety net.

Twelve infrared cameras (Qualisys AB, Gothenburg, Sweden) were used to record the three-dimensional location data of the markers at 300 Hz. Furthermore, the throws were recorded at 150 Hz by two perpendicular video cameras (Qualisys AB, Gothenburg, Sweden). Half of the infrared cameras were positioned on either side of the approach, thus forming an oval with the camera system positioned around 10 m in front and 2 m behind the foul line. One video camera was placed orthogonally to the approach, approximately two meters in front of the foul line. The second video camera recorded the athletes from behind, approximately 10 m behind the foul line. The calibrations average residual was 0.75 mm.

Following the warm-up routine of each athlete (approximately 30–45 min), every participant executed a minimum of three trials from their favored approach (average approach speed: 5.05 ± 0.62 ms^−1^). The javelin's release speed (*v*_0_) was used to choose the best three throws of each athlete for further analysis.

### 2.3 Data processing

**Figure 2:**
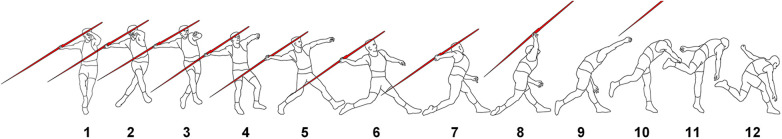
Time interval of a javelin throw from the push-off to the impulse step to the end of the deceleration movement. Other key points are the touchdown of the rear leg (4), touchdown of the bracing leg (6) and the release of the javelin (8).

Three crucial events were identified from the recorded video data prior to additional data analysis: (1) the touchdown of the rear leg, (2) the touchdown of the bracing leg, and (3) the javelin's release (Figure 2). In order to also consider the deceleration and follow-through phase, the time period of analysis was first set from the touchdown of the rear leg to 100 frames after release of the javelin A fourth order, zero-lag Butterworth filter was then used to filter the marker trajectories. Residual analysis was used to identify the cut-off frequencies (8–11 Hz) for each marker (18).

The kinematics and kinetics were calculated in Visual3D (Ver. 2024.03.1; C- motion, Germantown, USA) using a five-segment model of the javelin, right hand, right forearm, right upper arm, and thorax. While the wrist and elbow joint centers were determined as midpoints between the ulnar and radial styloid, and the medial and lateral humeral epicondyles respectively, the shoulder joint center was determined using the functional methods proposed by Schwartz and Rozumalski (19) and implemented into Visual 3D. Joint angles of the shoulder and elbow joint were calculated using Euler-/Cardan-sequences proposed by the International Society of Biomechanics (20). The position of the thorax in space was calculated via Cardan-sequence (ZYX) with respect to the laboratory coordinate system. Angular velocities and joint angular velocities were calculated as time derivatives of the respective rotation matrices.

The resultant joint forces (RJF) and torques (RJT) were calculated as external torques and forces by the top-down approach using Newton-Euler equations of motion and inverted to express them as internal torques and forces. This analysis is based on the assumption that the joint torque is generated by the muscles alone and that no translation is possible within the joint (21). De Leva's (22) body segment inertia parameters were used for invers-dynamic calculation, the center of mass (CoM) and the moments of inertia of the javelin were estimated with a reaction board and torsion pendulum (23). In order to calculate the kinetics before and after the release (REL), the computation was done twice. While the first included the javelin, the implement was removed for the second pass. All further data processing was done using custom written MATLAB (Ver. 23.2.0.25; The Mathworks Inc., Natick, MA, USA) script. The data of both modeling runs were connected at REL. Therefore, REL was determined more accurately by first calculating the center of mass of the javelin in each frame from its relative position to the attached markers. In the second step, the acceleration of the javelin's CoM was calculated as the second derivative of its position. REL was then determined as the last frame after the peak acceleration, where the Euclidean norm of the acceleration was greater than zero (24). Afterwards, the timepoint of the maximum internal rotation (tMIR) after REL was identified and the time range of analysis was set to tMIR + 0.1 s (12). As the joint angular velocities and RJT and RJF had been calculated in the global coordinate system, they were afterwards rotated into orthogonal coordinate systems, as proposed by Fleisig et al. (10).

From the calculated kinematics and kinetics, the EF between segments due to gravitational and inertial forces was computed by a segmental power analysis for all segments (hand, forearm, upper arm) at the connecting joints (wrist, elbow, shoulder). For the proximal and distal segments of the joints, the segment torque power (STP) and joint force power (JFP) were calculated as:

$$\mathrm{STP}=T_{\mathrm{ij}}{\dot{\theta }}_{\mathrm{ij}}$$

$$\mathrm{JFP}=F_{\mathrm{ij}}v_{j},$$

where $T_{\mathrm{ij}}$ and $F_{\mathrm{ij}}$ denote the RJT and RJF vector of the *i*^th^ segment of the *j*^th^ joint, respectively. ${\dot{\theta }}_{\mathrm{ij}}$ denotes the angular velocity of the *i*^th^ segment of the *j*^th^ joint and $v_{j}$ denotes the linear velocity of the *j*^th^ joint. While $\dot{\theta }$ is not necessarily the same for both segments connected by a joint, ***v*** is equal for both segments of a joint. Therefore, the JFP represents the rate of energy loss of one segment whose magnitude is equal to the rate of energy gain of the second segment at the same joint. In contrast, the STP reflects more than the rate of transfer of mechanical energy. Due to the different angular velocities of the segments, STP also contains mechanical energy generation and absorption (21). Table 1 shows how energy is transferred, absorbed, or generated at both segments depending on the STP. From the segmental power analysis, the net rate of energy transfer, rate of energy generation and energy absorption were calculated as outlined bin Table 1. The net rate of energy transfer was calculated as the sum of JFP and the part of STP, which reflects the rate of energy transfer. The resulting power-time curves were then integrated over time for the acceleration phase (until REL) and deceleration phase (after REL) in order to calculate the mechanical energy that was transferred, generated, or absorbed.

To quantify the demands placed on the shoulder by the resultant joint torques in the deceleration phase, the peak shoulder external rotation torque, the peak shoulder horizontal extension torque and the peak shoulder adduction torque were identified after REL.

**Table 1:** Calculation of the transfer, generation and absorption of mechanical energy depending on the magnitude and sign of the STP of both segments of a joint (2, 12, 21).

		Generation	Absorption	Transfer
Same sign				
Both positive		To proximal segment at *T_p_*$\dot{\theta }$*_p_* To distal segment at *T_d_*$\dot{\theta }$*_d_*	0	0
Both negative		0	From proximal segment at *T_p_*$\dot{\theta }$*_p_* From distal segment at *T_d_*$\dot{\theta }$*_d_*	0
Opposit sign				
|STP_p_| >	|STP_d_|			
+	-	To proximal segment at *T_p_*($\dot{\theta }$*_p_*-$\dot{\theta }$*_d_*)		To proximal segment at *T_d_*$\dot{\theta }$*_d_*
-	+	0	From proximal segment at *T_p_*($\dot{\theta }$*_p_*-$\dot{\theta }$*_d_*)	To distal segment at *T_d_*$\dot{\theta }$*_d_*
|STP_p_| <	|STP_d_|			
+	-		From distal segment at *T_d_*($\dot{\theta }$*_d_*-$\dot{\theta }$*_p_*)	To proximal segment at *T_p_*$\dot{\theta }$*_p_*
-	+	To distal segment at *T_d_*($\dot{\theta }$*_d_*-$\dot{\theta }$*_p_*)	0	To distal segment at *T_p_*$\dot{\theta }$*_p_*

STP*_p_*_,_ segment torque power of the proximal segment; STP*_d_*, segment torque power of the distal segment; *T_p_*, proximal joint torque vector; *T_d_*, distal joint torque vector; $\dot{\theta }$*_p_*, angular velocity vector of the proximal segment; $\dot{\theta }$*_d_*, angular velocity vector of the distal segment.

Variables of interest, which presumably could influence resultant joint torques were identified in the acceleration phase along with the release velocity. The variables analyzed are segments and joints that are in direct close range to the shoulder joint and thus can have a direct influence on it. Therefore, the forward lean of the thorax (the angle between the frontal plane of the thorax and vertical axis of the global coordinate system), the shoulder external rotation, the shoulder horizontal extension and elbow angle were calculated at REL. Furthermore, the maximum thorax angular velocity about its longitudinal and sagittal axis, maximum shoulder internal rotation and horizontal flexion velocities, and the maximum elbow extension velocity were calculated. Besides the kinematics, the energy flow in the acceleration was quantified. Therefore, the kinetic energy of the javelin at release, the peak rate of energy transfer from proximal to distal, the peak rate of energy generation at the shoulder, the amount of energy transferred from proximal to distal and the amount of energy generated at the shoulder were computed.

### 2.4 Statistics

To investigate the influence of the kinematics and energy flow during the acceleration phase, specifically at the timepoint of release, on the resultant joint torques of the shoulder during the deceleration phase, regularized regression models were fitted. As the normalization of the kinetic variables by body mass or body mass*body height could lead to distortions in prediction, we decided to not normalize our kinetic data and instead to include mass and height as predictor variables (25). To eliminate magnitude influences on regressor shrinkage due to different measurement scales, predictor variables were standardized. To combine the advantages of ridge regression and the least absolute shrinkage and selection operator (LASSO), the Elastic Net adjustment for regularized regression was used (26). As the focus lied on variable selection, the alpha-coefficient was set to 0.75. Each regression was cross-validated 10 times over 100 *λ*-values, which controlled severity of the penalty in regularized regression (26, 27). For each peak resultant joint torque in the deceleration phase two regularized regression models were calculated, whereby each dependent variable was attempted to be explained by the kinematic or EF variables. The model that best predicts the dependent variable was chosen using the smallest mean squared error (MSE) which was calculated for the model of each *λ*-value. This ensured the best accuracy for each model (26). The unstandardized regression coefficients were reported for the standardized predictors.

## 3 Results

**Figure 3:**
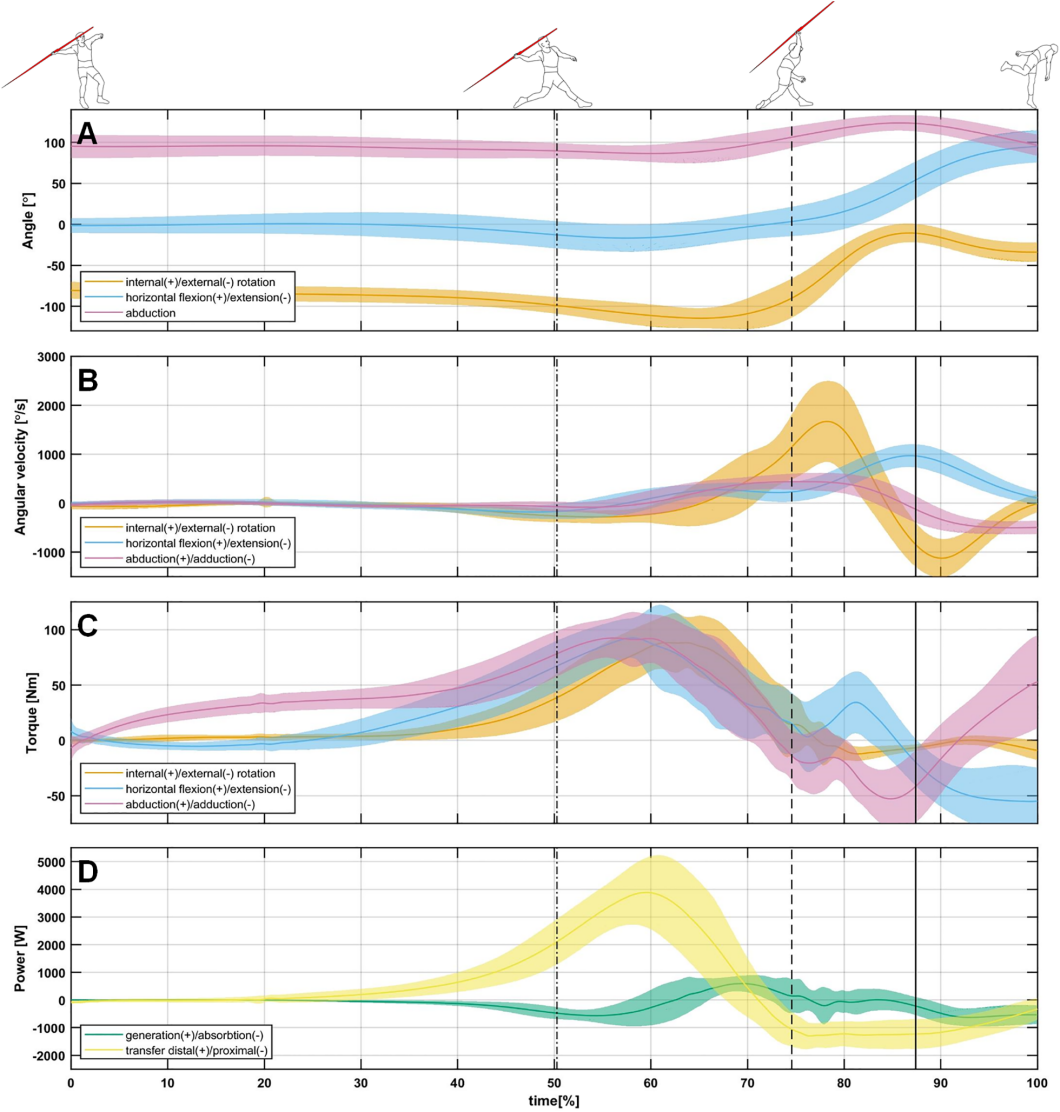
Time series of the shoulder joint angles **(A)**, shoulder joint angular velocities **(B)**, shoulder joint torques **(C)**, and rates of energy transfer and generation and absorption at the shoulder **(D)** from the touchdown of the rear leg to the timepoint of maximal internal rotation + 0.1 s, as relative time. The dash-dotted line indicates the touchdown of the bracing leg, the dashed line indicates the release, and the continuous line represents the timepoint of maximum internal rotation.

The mean release velocity of the investigated athletes reached *v*_0_ = 21.48 ± 1.23 ms^−1^. The movement of the throwing arm reached a mean of 8.94 ± 10.32° external rotation and a mean of 82.97 ± 6.39° horizontal flexion in the deceleration (Figure 3A). In this phase the external joint loads peaked at a mean shoulder horizontal extension torque of 66.56 ± 32.54 Nm, while the mean peak shoulder external rotation torque reached 23.40 ± 8.48 Nm (Figure 3C). The mean peak adduction torque was calculated with a value of 63.95 ± 19.11 Nm. The mean peak rate of energy absorption at the shoulder reached 924 ± 383 W, while energy was transferred with a mean peak rate of 1,621 ± 413 W from distal to proximal (Figure 3D). Thereby, a mean energy of 49.6 ± 17.4 J was absorbed at the shoulder, while a mean energy of 166.5 ± 53.1 J was transferred from distal to proximal.

The regularized regression models were able to predict the resultant joint torques placed on the shoulder based on kinematics (Table 2) and EF (Table 3). While the shoulder external rotation and horizontal extension torque could be predicted by kinematics and energy flow, the shoulder adduction could only be predicted by kinematics. The number of predictors varied between 6 and 9 (kinematics) and 4–6 (energy flow).

**Table 2:** Mean and standard deviation (Std) of the kinematic predictors of the acceleration phase, determined with elastic Net regularized regression, as well as the unstandardized regression coefficients for the models predicting peak shoulder external rotation torque (T^ER^), peak shoulder horizontal extension torque (T^hExt^), and the peak shoulder adduction torque (T^ADD^).

			Mean Std	T^ER^	T^hExt^	T^ADD^
	Intercept			−18.82	28.67	−101.16
	Mass	[kg]	93.34 ± 10.3	0.078		0.316
	Height	[m]	1.89 ± 0.1		3.121	13.609
	Release speed	[ms^−1^]	21.48 ± 1.2		1.319	3.599
Angle at release	Thorax forward tilt	[°]	1.54 ± 15.1	−0.033		0.370
	Shoulder external rotation	[°]	92.98 ± 12.1	−0.175	−0.011	
	Shoulder hor. Flexion	[°]	1.99 ± 14.4	0.116	0.283	−0.024
	Elbow flexion	[°]	29.05 ± 6.0			
Maximum angular velocity	Shoulder internal rotation	[°/s]	2,183 ± 504.6	0.004		
	Shoulder horizontal flexion	[°/s]	1,055 ± 162.6	0.007		0.009
	Elbow extension	[°/s]	308 ± 144.3	0.006		0.011
	Thorax rotational	[°/s]	823 ± 139.0		−0.011	−0.007
	Thorax forward tilt	[°/s]	275 ± 84.4		0.042	0.088

**Table 3:** Mean and standard deviation of the energy flow predictors from the acceleration phase determined with elastic Net regularized regression, as well as the unstandardized regression coefficients for the models predicting peak shoulder external rotation torque (T^ER^), peak shoulder horizontal extension torque (T^hExt^) and the peak shoulder adduction torque (T^ADD^).

		Mean Std	T^ER^	T^hExt^	T^ADD^
Intercept			−283.07	17.01	61.70
Mass	[kg]	93.3 ± 10	0.40		
Height	[m]	1.9 ± 0	121.03	17.41	
P^P^^→D^	[W]	4,466 ± 1,071	−0.01		
P^gen^	[W]	785 ± 168	0.02	−0.01	
E^P^^→D^	[J]	392 ± 73			
E^gen^	[J]	32 ± 9	0.23	−0.89	
E^Kin^	[J]	193.4 ± 22	0.25	0.27	

P^P→D^ peak rate of energy transfer from proximal to distal; P^gen^ peak rate of energy generation; E^P→D^ energy transferred from proximal to distal; E^gen^ energy generated; E^Kin^ kinetic energy of the javelin at release.

## 4 Discussion

The aim of the study was to investigate (i) how the remaining mechanical energy of the throwing arm is dissipated through gravitational and inertial forces via the shoulder, (ii) which resultant joint torques are placed on the shoulder and (iii) how resultant joint torques at the shoulder are influenced by the kinematics and energy flow of the acceleration phase. This study is therefore the first study trying to establish a relation between the parameters of the release phase and the resultant joint torques on the shoulder in the deceleration phase.

The results show that a large part of the energy that must be dissipated from the throwing arm is transferred back to the upper body through the shoulder. Interestingly, the return of energy begins even before the javelin has left the hand (Figure 3D). The amount of energy that was returned is more than the values reported by Wasserberger et al. (12), even though the athletes had release velocities 10 m/s^−1^ higher than the throwers in this study. However, it must be taken into account that the athletes investigated by Wasserberger et al. (12) had significantly less mass (74.1 ± 4.2 kg) than the athletes examined here. Relative to body mass, the energy transferred backwards is comparable between both studies. This results in 1.74 ± 0.82 J/kg and 1.8 ± 0.57 J/kg between pitching and javelin throwing, respectively. While these values are comparable, it must be considered that the javelin throwers return an equal amount of energy mainly due to a higher mass of the arm, while the baseball players achieve these amounts of energy mainly due to higher speeds. When one considers the amount of energy absorbed, this ratio changes, the baseballers absorb significantly higher amounts of energy in total (79 ± 36 J vs. 49.6 ± 17.4 J) and therefore also relative to body mass (1.01 ± 0.45 J/kg vs. 0.53 ± 0.19 J/kg). Due to the same relative amount of energy that is returned proximally, it can be hypothesized that the amount of energy returned in a given time interval is limited and thus the athletes in baseball have to compensate by absorbing energy (28). However, one could also assume that the javelin throwers have better muscular stabilization of the shoulder due to their higher training age and are therefore better able to redirect the remaining energy. In this context, Barfield et al. (29) have shown that athletes with a more muscularly secured shoulder exhibit higher rates of energy transfer in the deceleration phase. Since the absorption of mechanical energy requires eccentric contraction, but the transfer of mechanical energy does not, the transfer of energy backwards can be considered less stressful. It has also been shown for the acceleration phase that the transfer of mechanical energy is less stressful than its generation (2, 13). However, this is yet to be examined in more detail. When comparing energy transfer of the deceleration to the acceleration phase (2), a significant lower transfer of energy can be noted. But it must be remembered, that (a) a large amount is transferred to the implement and therefore does not have to be transferred backwards and (b) at the end of the analyzed time period the arm is still not at rest and thus contains kinetic energy that must be dissipated.

Compared to the resultant joint moments in baseball, the calculated RJT of this study are clearly lower (5, 10). However, the distribution of joint torques is similar, while the horizontal extension and adduction show the highest torques, the external rotation torque is well below them. While Köhler & Witt (2) calculated even higher joint torques for the acceleration phase which they primarily attributed to the higher mass of the javelin, no implement was present in the deceleration phase. The increased muscular demands could therefore be attributed to the higher speeds in baseball, which must be slowed down after release. The regression models that use the kinematics as predictors also show that the release speed is an influencing factor for the resultant joint torques in the deceleration phase. The higher the release velocity, the higher the following resultant joint torques on the shoulder. This has already been proven for the acceleration phase in baseball and javelin throwing (2, 30). However, it can also be demonstrated that the resultant joint torques on the shoulder are influenced by other kinematic variables of the acceleration phase. The external rotation and horizontal flexion of the shoulder at the time of release influence the external rotation and horizontal extension torque. The greater the external rotation, the lower the resultant joint torque, and the bigger the horizontal flexion the higher the resultant joint torque during deceleration. The greater external rotation gives athletes a longer braking path, which means they can apply less torque over a longer period and still stop the arm, wheras a higher horizontal flexion would decrease the stopping distance. Furthermore, it can be seen, at least for the external rotation torque, that a more forward tilted upper body at the time of release reduces the RJT. As the athletes must accelerate the javelin along its longitudinal axis and at the same time achieve an optimal release angle, a further forward movement of the upper body means that the athletes must remain in external rotation in order to do so. Therefore, the extended forward tilt of the upper body could influence the external rotation and thus work towards reducing the resultant joint torque during deceleration. This may imply that an increased forward tilt of the thorax would not only be a prerequisite for achieving high release speeds (2) but could also be useful in preventing injuries. However, it must also be further examined as to whether the reduction of one resultant joint torque does not result in an increase in another torque. For example, the regression models show that increasing the upper body forward tilt reduces the external rotation torque but increases the adduction torque at the same time. For the horizontal extension angle at release, an opposite behavior of both torques can be shown, as the angle increases, the external rotation torque increases and the adduction torque decreases.

Furthermore, the resultant joint torques are influenced by the peak angular velocities of the acceleration phase. Thus, an increase in angular velocities leads to an increase in the resultant joint torques in the deceleration phase as these higher velocities must be stopped. However, the high angular velocities at the shoulder are also a prerequisite for high release speed (9). On the other hand, the peak angular velocity of the upper body about its longitudinal axis can reduce resultant joint torques. As Aguinaldo & Escamilla (31, 32) reported, the rotational motion of the thorax is an important contributor to energy transfer across the shoulder. A faster rotating thorax may lead to smaller horizontal flexion angles (or higher horizontal extension angles) at release and therefore reduce the resultant joint torques, as stated for the horizontal flexion angle before. This is partly confirmed by the regression models of kinetics. A higher transfer of mechanical energy via the shoulder to the distal segment leads to a reduction of the resultant joint torques, but not for the horizontal extension torque. As it has already been proven for the acceleration phase, the generation of energy has a negative effect on the resultant joint torques (13, 14), at least in the case of external rotation, while the horizontal extension shows a reversed relationship. This correlation should be examined more closely, as it cannot be explained by the authors at this point. The kinetic energy of the javelin at the time of its release is, like the release speed of the implement, associated with an increase in the resultant joint torques in the deceleration phase. Performing more work on the implement therefore also requires more energy from the segments, which can be achieved by increasing their velocity. However, the authors would also have expected the amount of transferred energy to be a predictor of the resultant joint torques in the deceleration phase, as this is one of the most important factors for increased release speed and could therefore also influence the demands on the joint (2, 12). When comparing the resultant joint torques between the acceleration and deceleration phase one can note differences in the magnitudes of the resultant joint torques. While in the acceleration phase a broad variety of muscles is active to stop external rotation by eccentric contraction and accelerating the arm due to concentric contraction, the rotator cuff is mainly active to control humeral head positioning. In the deceleration phase the muscles of the rotator cuff have to contract to resist distraction, horizontal adduction, and internal rotation of the shoulder (33). These additional tasks and the eccentric contraction to stop the internal rotation, could possibly explain why the rotator cuff is exposed to a greater risk of injury during the deceleration phase, even though the joint torques are lower. The reduction of the resultant joint torques could by adjusting the body positioning due to technical improvement is therefore crucial to minimize the damends placed on the joint and therefore reduce the risk of injury either due to repeated stress or singular events.

## 5 Limitations

The following limitations should be considered when evaluating the current study's findings. First and foremost, the sample size needs to be considered. From a statistical point of view, the group size is relatively small. However, if you look at the athletes’ personal bests and the fact that this is not a competition investigation, there are no comparable investigations to date. Furthermore, the study shows results that are consistent with other findings. We therefore assume that the results have a practical relevance despite the small sample size.

Second, in contrast to competition results, the release velocities were comparatively low. There could be several causes for this. (a), it is important to note that the investigation was completed several months prior to the competitive season. (b), the investigation was conducted indoors, which contrasts with the requirements of a competition.

Third, although great importance was placed on the exclusion of predictors in the regularized regression estimation with a value of *α* = 0.75, the regression models still contain a relatively large number of variables. However, several relevant (practical and clinical) results could be found. Despite this, more research is needed to better understand the deceleration movement, the loads occurring in this phase and how they are linked to the acceleration phase.

Ultimately, there are several restrictions associated with motion capture and multi-body modeling. Errors can occur when calculating joint centers, due to marker motion, and the estimation of body segment inertia parameters. Nevertheless, every effort was made to reduce their impact as much as possible within the selected approaches.

## 6 Conclusion and perspectives

Our study is the first to investigate the resultant joint torques and energy flow in the deceleration phase of the javelin throw and how they are linked to the energy flow and kinematics of the acceleration phase. We were able to show that energy flow in the acceleration phase and the resultant joint torques in the deceleration phase are linked, but that at the same time, the demands can be altered by changed joint angles at the point of release. Therefore, it is possible to optimize the movement regarding load minimizing and performance maximizing at the same time. However, the results only represent a first approach. More studies are needed to understand the mechanisms of the kinematic chain and the underlying mechanical patterns in the acceleration and the deceleration phase and their linkage. This improved understanding could lead to better technical preparation for athletes and thus contribute to injury prevention.

## Data Availability

The raw data supporting the conclusions of this article will be made available by the authors, without undue reservation.
